# Effect of the German tonsillitis guideline on indication for tonsil surgery in patients with recurrent acute tonsillitis: a population-based study

**DOI:** 10.1038/s41598-023-44661-y

**Published:** 2023-10-17

**Authors:** Valerie Betz, Daniel Boeger, Jens Buentzel, Kerstin Hoffmann, Peter Jecker, Holger Kaftan, Andreas Mueller, Gerald Radtke, Katharina Geißler, Orlando Guntinas-Lichius

**Affiliations:** 1 Department of Otorhinolaryngology, Jena University Hospital, Jena, Germany; 2 Department of Otorhinolaryngology, Zentralklinikum, Suhl, Germany; 3 Department of Otorhinolaryngology, Südharz-Krankenhaus gGmbH, Nordhausen, Germany; 4 Department of Otorhinolaryngology, Sophien/Hufeland-Klinikum, Weimar, Germany; 5 Department of Otorhinolaryngology, Klinikum Bad Salzungen, Bad Salzungen, Germany; 6 Department of Otorhinolaryngology, Helios-Klinikum, Erfurt, Germany; 7 Department of Otorhinolaryngology, SRH Wald-Klinikum, Gera, Germany; 8 Department of Otorhinolaryngology, Ilm-Kreis-Kliniken, Arnstadt, Germany; 9 Department of Otorhinolaryngology, Jena University Department, Am Klinikum 1, 07747 Jena, Germany

**Keywords:** Oral diseases, Pain

## Abstract

Evidence-based indication for tonsil surgery in patients with recurrent acute tonsillitis (RAT) is an ongoing matter of debate. Since introduction of the German tonsillitis guideline in 2015, the indication criteria for tonsil surgery have become much stricter. It is unclear, if this has changed the indication policy. A retrospective population-based study was performed including all 1398 patients with RAT admitted for tonsil surgery in all Thuringian hospitals in 2011, 2015, and 2019. Changes over the years concerning patients’ characteristics, number of tonsillitis episodes in the last 12 months treated with antibiotics (T12), and decision for tonsillectomy or tonsillotomy were analyzed using univariable and multivariable statistics. The surgical rates decreased from 28.56/100,000 population in 2011 to 23.57 in 2015, and to 11.60 in 2019. The relative amount of patients with ≥ 6 T12 increased from 14.1% in 2011 over 13.3% in 2015 to 35.9% in 2019. Most patients received a tonsillectomy (98% of all surgeries). Decision for tonsillotomy was seldom (1.2%). Multinomial logistic regression analysis with the year 2011 as reference showed that compared to the year 2015, the age of the patients undergoing surgery increased in 2015 (Odds ratio [OR] = 1.024; 95% confidence interval [CI] = 1.014–1.034; p < 0.001), and also in 2019 (OR 1.030: CI 1.017–1.043; p < 0.001). Compared to 2011, the number T12 was not higher in 2015, but in 2019 (OR 1.273; CI 1.185–1.367; p < 0.001). Stricter rules led to lower tonsil surgery rates but to a higher proportion of patients with ≥ 6 T12 before surgery. Tonsillectomy remained the dominating technique.

## Introduction

Tonsillitis is a very common condition with an estimated incidence in general practice of 37–100 per 1000 population a year^1^. The incidence of RAT, i.e., how many tonsillitis patients develop a RAT, is less clear. A population-based twin study reported a lifetime prevalence of RAT of 11.7% (higher for females with 14.1% compared to males with 8.8%)^2^. The problem is that RAT and also the cut-off for tonsil surgery are arbitrarily and inhomogeneously defined by the number of episodes in one year. Most cited are the Paradise criteria saying that indication for tonsillectomy is given if ≥ 7 physician-diagnosed episodes occurred in the last 12 months, or ≥ 5 episodes in last 24 months, or ≥ 3 episodes in last 36 months. This recommendation is based on results of a randomized controlled study in children 3–15 years of age, i.e., adult patients were not included^3^. In a subsequent study, analyzing a more moderate cut-off (3–5 episodes in 12 months), tonsillectomy was not better than conservative management to reduce sore throat episodes in the following 12 months^4^. The Scottish Intercollegiate Guidelines Network (SIGN) guideline is probably the oldest guideline to recommend tonsillectomy based on the number of sore throat episodes due to acute tonsillitis. Due to the actual SIGN guideline, a tonsillectomy is recommended if ≥ 5 episodes in each of the preceding two years or ≥ 3 episodes in each of the preceding three years occurred^5^. The German guideline was introduced in 2015 and stated that tonsillectomy can reduce sore throat episodes for the following 12 months is moderate for children and low for adults^6^. Therefore, the cut-off for the indication for tonsillectomy was defined very strictly and equally for children and adults: < 3 episodes in 12 months = no indication; 3–5 episodes in 12 months = might be an indication, if more episodes occur in the next 6 months; ≥ 6 episodes in 12 months = indication. Furthermore, the episodes must have been diagnosed by a physician and have been treated by antibiotics. Finally, the guideline recommended in such a situation either a tonsillectomy or a tonsillotomy as equivalent tonsil surgery techniques to treat the RAT.

Like in many other countries, the annual tonsil surgery rates have declined in Germany in the last decades. It is suspected that this is due to the introduction of the tonsillitis guideline and the mentioned cut-off values. Between 2005 and 2017, 1,313,449 tonsil surgeries were registered in Germany. There was a decrease in the rate of tonsillectomy from 92 in 2005 to 43/100,000 population in 2017 in Germany^7^. Hence, tonsil surgery still is one of the most frequent head and neck surgeries, especially in children and young adults.

In this regard, it is of interest to analyze if the tonsillitis guideline has changed the indication to treat RAT by tonsil surgery in the daily clinical routine. Thuringia is a territorial state in Germany with approximately 2.2 million inhabitants. There are only eight hospitals with departments of otorhinolaryngology in Thuringia. The departments of otorhinolaryngology have built a network primarily to improve health services research in the field of otorhinolaryngology. Use of this network provided an ideal platform for a population-based analysis of patients admitted with RAT for tonsil surgery before and after the introduction of the tonsillitis guideline in 2015. Changes in the characteristics of the RAT patients and indication behavior of the otolaryngologists between the year 2011, 2015, and 2019 were the focus of the present study.

## Methods

### Study design and setting

The institutional ethics committee of the Jena University Hospital, Jena, Germany, approved the study protocol for a retrospective data collection (no. 2726-12/09; no. 4370-03/15). The study followed the ethical standards outlined in the Declaration of Helsinki and was carried out in accordance with all other relevant guidelines and regulations. Only anonymized data were analyzed. Therefore, the ethics committee waived the need for written informed consent. A standardized retrospective analysis was performed in all eight Thuringian hospitals with a department of otolaryngology (in alphabetic order: Arnstadt, Bad Salzungen, Erfurt, Gera, Jena, Nordhausen, Suhl, Weimar). All patients were selected who were coded for RAT (J35.0 due to the International Classification of Diseases [ICD], 10^th^ revision, German modification; ICD-10-GM) and who were treated in the years 2011, 2015, and 2019. The year 2015 was chosen because the German clinical guideline for the treatment of tonsillitis was launched in 2015^6^. The years 2011 and 2019 were chosen to guarantee a sufficient distance (4 years) before and after introduction of the guideline. Initially, the data set contained 3007 patients. 102 patients with tonsil cancer, 383 patients with confirmed unilateral peritonsillar abscess, 398 patients with acute tonsillitis (without recurrent episodes, as explicitly noted in the patient chart), 170 patients with primary diagnosis of obstructive sleep apnea, and 35 patients with mononucleosis were excluded. 2009 patients with RAT remained in the database. Out of these patients, 611 patients were excluded because the number of tonsillitis episodes in the last 12 months was not documented. Hence, the final study cohort included 1398 patients. To rule out an important selection bias, the included RAT patients with documented episodes were compared with the RAT patients without documentation (Supplement Table S1). The number of patients without documentation decreased over the years. In the cohort without documentation, the rate of patients not undergoing tonsil surgery, the rate of tonsillotomy in relation to tonsillectomy, the rate of additional adenoidectomy, and the age were higher (all p < 0.001).

A retrospective search of the patients’ charts was performed. The following variables were obtained: age, sex, comorbidity, physical examinations, medical treatment and surgical procedures related to the RAT. The number of tonsillitis episodes treated with antibiotics (regardless of which medical discipline prescribed them) in the last 12 months was counted. This number was highly relevant, because the guideline recommends a tonsillectomy or a tonsillotomy as an option for treatment of RAT only if the patient had ≥ 6 episodes of a tonsillitis (1) diagnosed by a physician and (2) treated with antibiotics^6^. Another special feature of the guideline is that it recommends equally tonsillectomy and tonsillotomy if the tonsils are larger than Brodsky grade 1^8^. Tonsillotomy is defined as a restriction of the resection of the tonsillar tissue medial to the palatal arches, i.e., the parts inside the palatal arches are not resected^6^. Hence, tonsillotomy is not the same as intracapsular tonsillectomy. Intracapsular tonsillectomy is not recommended in the guideline.

Before analysis, the data was blinded with respected to the treating hospital.

The epidemiological calculations for the incidence of tonsil surgery (surgical rate) per 100,000 population were based on the annual mean number of habitants in Thuringia in 2011, 2015, and 2019. Population numbers of the online database of the Thuringian State Office for Statistics (www.tls.thueringen.de) were used.

### Statistical analysis

Participants’ characteristics and outcome variables were analyzed with IBM SPSS statistics software (Version 28.0.0.0) for medical statistics. Data are presented as mean ± standard deviation (SD) if not otherwise indicated. The chi-square test was used to compare nominal data of two independent subgroups. Fisher’s exact test was used to compare ordinal data of ≥ 2 independent subgroups. The Mann–Whitney U-test was used to compare scaled data of two independent subgroups. Multinomial logistic linear regression models were generated to determine odds ratios (OR) and 95% confidence intervals (CI) for the analysis of patients’ characteristics for the different years (2011, 2015, and 2019). The reference year was 2011. An OR indicates the chance that a patient’s characteristic is present in a subgroup of patients in the years 2015 or 2019. Patients’ characteristics for regression analysis were derived from those variables that were significant in preliminary univariate analyses (p < 0.05). In general, nominal p values of two-tailed tests are reported.

## Results

### Study participants

Patients’ characteristics are summarized in Table 1. Eight-hundred and fifty-one (851) female and 547 male participants (female to male ratio: 1.6:1) were analyzed. The mean age was 23.8 ± 12.4 years. Only a few patients (1.1%) did not undergo tonsil surgery. Most patients were treated by bilateral tonsillectomy (98.2%). The number of tonsillotomies for RAT was low with n = 25 (1.8%). The number of tonsil surgeries decreased by 59% from 2011 (n = 626) to 2019 (n = 254).

**Table 1:** Patients’ characteristics of all patients (n = 1398).

Parameter	All		2011		2015		2019		p
	n	%	n	%	n	%	n	%	
All	1398	100	626	100	518	100	254	100	
Gender									0.550
Male	547	39.1	242	38.7	198	38.2	107	42.1	
Female	851	60.9	384	61.3	320	61.8	147	57.9	
Reason against surgery									**0.027**
Patient had surgery	1383	98.9	625	99.8	510	98.5	248	97.6	
Contraindication	1	0.1	0	0	0	0	1	0.4	
Patient declined surgery	1	0.1	0	0	1	0.2	0	0	
No indication	13		1	0.2	7	1.4	5	2.0	
Setting									0.118
Inpatient	1389	99.4	625	99.8	513	99.0	251	98.8	
Outpatient	9	0.6	1	0.2	5	1.0	3	1.2	
Surgery									**0.007**
Yes	1383	98.9	625	99.8	510	98.5	248	97.6	
No	15	1.1	1	0.2	8	1.5	6	2.4	
Type of tonsil surgery*									0.056
Tonsillectomy	1358	98.2	617	98.7	502	98.4	239	96.4	
Tonsillotomy	25	1.8	8	1.3	8	1.6	9	3.6	
Additional adenoidectomy*									0.297
No	1381	99.9	623	99.7	510	100	248	100	
Yes	2	0.1	2	0.3	0	0	0	0	
Side of surgery*									**0.026**
One side	16	1.2	5	0.8	4	0.8	7	2.8	
Both sides	1367	98.8	620	99.2	506	99.2	241	97.2	

	Mean ± SD	Mean ± SD	Mean ± SD	Mean ± SD					
Age, years	23.8 ± 12.4	22.1 ± 12.6	25.3 ± 12.3	25.0 ± 11.5	** < 0.001**				
Tonsillitis episodes last 12 months, n = 1383	3.98 ± 2.01	3.82 ± 1.89	3.78 ± 1.89	4.78 ± 2.30	** < 0.001**				
Treatment duration, days*	6.0 ± 1.4	6.2 ± 1.4	6.0 ± 1.4	5.7 ± 1.5	** < 0.001**				

Significant p-values (p < 0.05) in bold.

*SD* standard deviation.

*Only of the patients who underwent surgery.

### Surgical rates over the years

**Figure 1:**
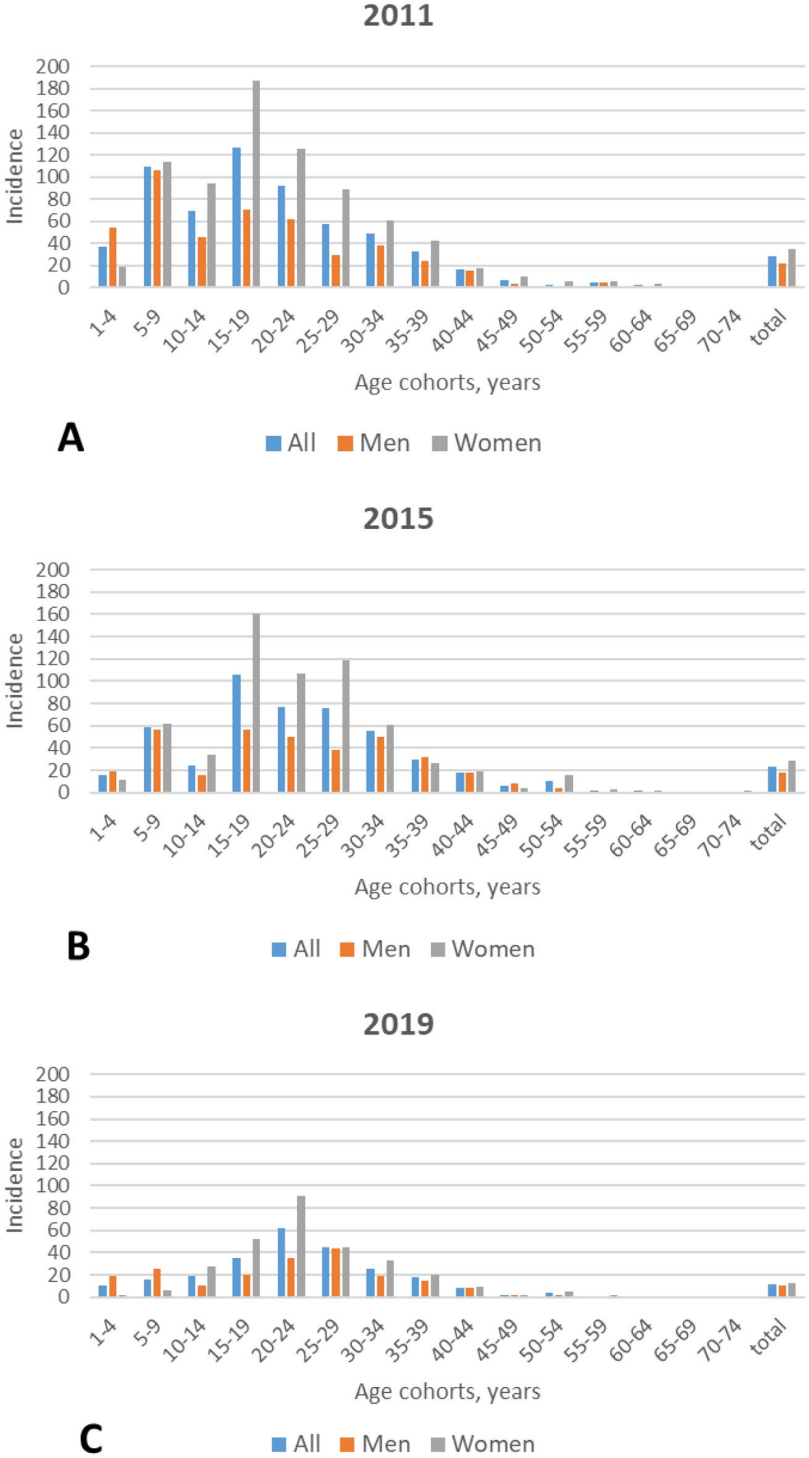
Incidence of tonsil surgery (surgical rate) per 100,000 population for all patients (blue), and separately for men (red) and women (grey) for the different age cohorts in Thuringia, Germany. (**A**) 2011, (**B**) 2015, (**C**) 2019.

Thuringia had 2,188,474 (male: 1,076,203; female: 1,112,271), 2,163,737 (male: 1,069,035; female. 1,094,702), and 2,138,262 (male: 2,138,262; female: 1,058,405) habitants in 2011, 2015, and 2019, respectively. The surgical rates decreased from 28.56/100,000 in the year 2011 to 23.57/100,000 in 2015, and to 11.60/100,000 in 2019 (Table 2). Beyond an age of 10 years, the surgical rates were always higher for female than for male patients. The age cohort with the highest surgical rate became older over time, both for male and female patients (Fig. 1). Overall, the highest surgical rate in 2011 was 186.57/100,000 for women aged 15–19 years. The highest rate in 2015 was also for women aged 15–19 years with 160.39/100,000. The highest rate in 2019 was then for women aged 20–24 years with 91.06/100,000.

**Table 2:** Surgical rates per 100,000 population in Thuringia depending on patient’s age and number of tonsillitis episodes.

Age cohort (years)	2011			2015			2019		
	All	Men	Women	All	Men	Women	All	Men	Women
1–4	37.40	54.72	19.18	15.56	19.53	11.39	10.95	19.23	2.25
5–9	109.59	**105.68**	113.70	58.82	56.14	61.65	16.15	25.21	6.63
10–14	69.32	45.88	93.96	24.71	16.06	33.80	19.29	11.00	28.13
15–19	**126.72**	70.07	**186.57**	**106.19**	**56.69**	**160.39**	35.42	19.74	52.46
20–24	92.36	62.41	125.47	77.14	49.84	107.17	**61.59**	35.64	**91.06**
25–29	57.08	29.81	89.09	75.79	38.50	118.74	44.32	**43.87**	44.83
30–34	48.73	38.10	61.04	55.08	49.88	61.11	25.92	19.62	32.97
35–39	32.54	23.99	42.41	29.36	32.13	26.21	17.73	15.31	20.47
40–44	16.22	15.02	17.51	18.34	17.97	18.77	8.43	7.95	8.97
45–49	6.94	4.21	9.76	6.28	8.54	3.88	1.49	1.41	1.58
50–54	2.72	0	5.49	10.07	4.20	16.05	3.65	2.39	4.96
55–59	5.05	4.50	5.59	2.25	1.13	3.37	1.07	0	2.15
60–64	2.13	0	4.17	1.75	1.19	2.30	1.17	1.19	1.16
65–69	0	0	0	0	0	0	0	0	0
70–74	0.64	1.41	0	0.76	0	1.39	0	0	0
Total	28.56	22.39	34.52	23.57	18.43	28.59	11.60	10.11	13.06
Episodes**									
< 3 episodes	6.08	4.92	7.19	4.85	4.58	5.12	1.78	1.70	1.85
3–5 episodes	**18.46**	**13.94**	**22.84**	**15.57**	**11.69**	**19.37**	**5.66**	**4.82**	**6.48**
≥ 6 episodes	4.02	3.53	4.50	3.14	2.15	4.11	4.16	3.59	4.72

*Highest values per year and gender are shaded in bold.

**Last 12 months.

### Number of tonsillitis episodes in last 12 months

**Figure 2:**
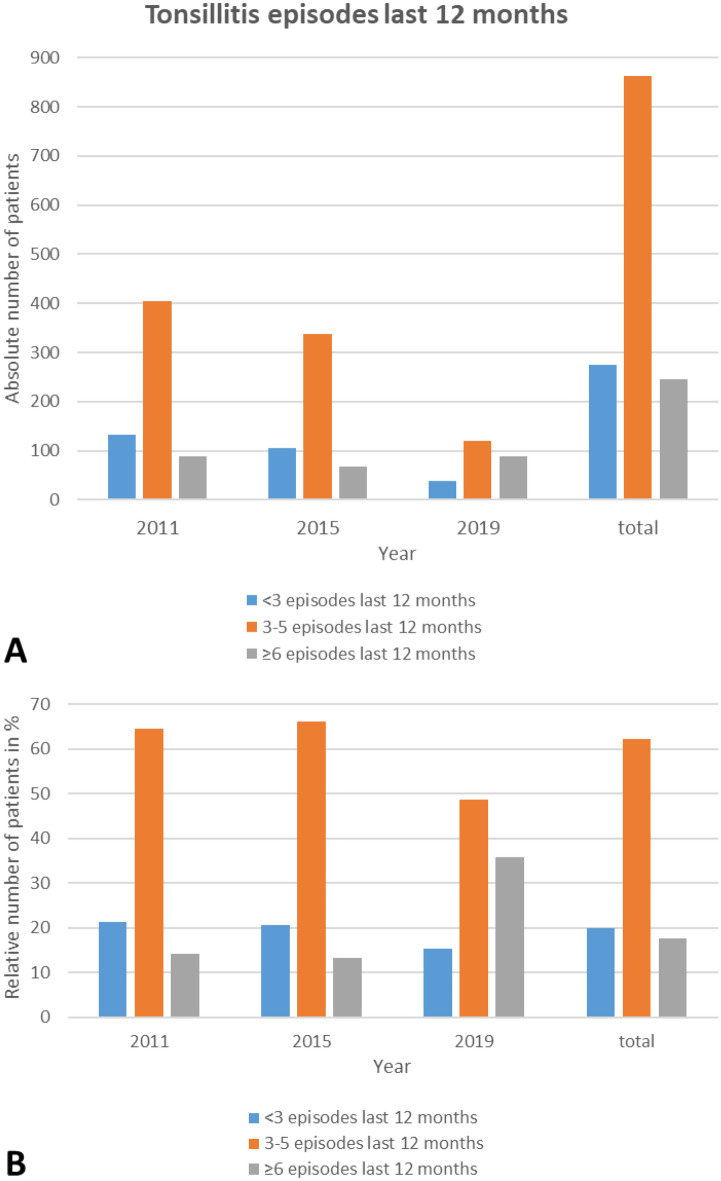
Tonsillitis episodes per patient in the last 12 months treated with antibiotics for the years 2011, 2015, and 2019. (**A**) Absolute number of patients. (**B**) Relative number of patients.

**Figure 3:**
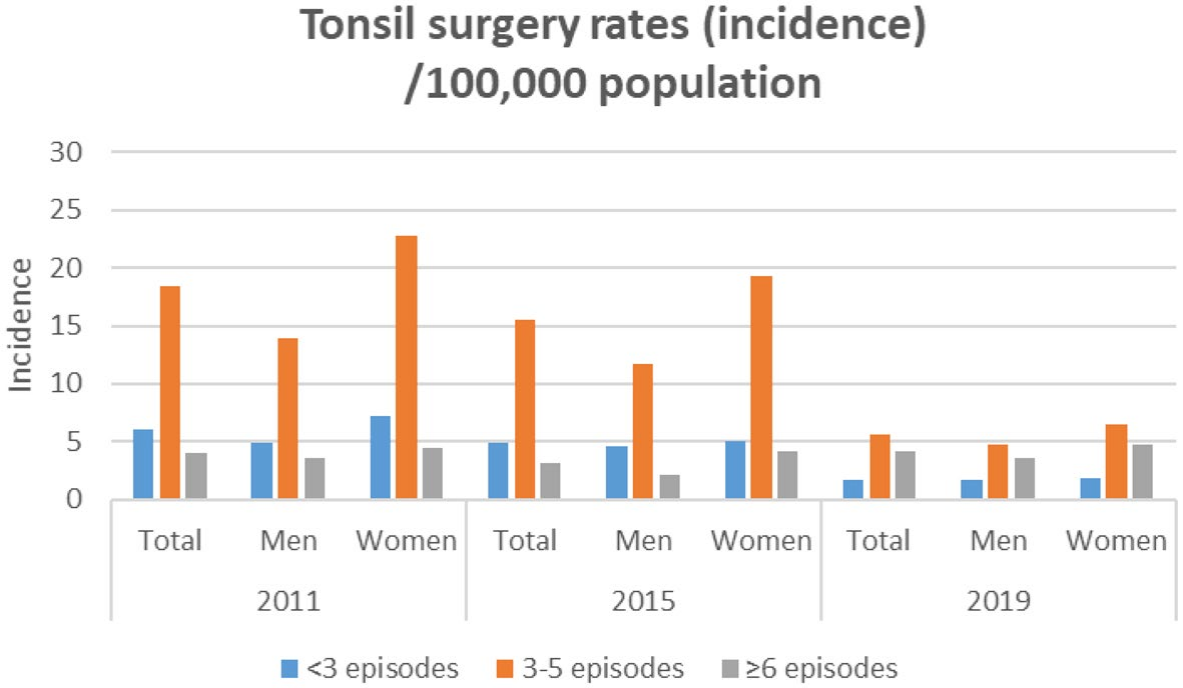
Incidence of tonsil surgery (surgical rate) per 100,000 population for all patients, men, and women for 2011, 2015, and 2019 separately for < 3 episodes (blue), 3–5 episodes (red), and ≥ 6 episodes (grey) of tonsillitis in the last 12 months prior to surgery.

The number of tonsillitis episodes in the last 12 months per patient treated with antibiotics before tonsil surgery changed over time (Fig. 2; Table 3). In all three selected years, the majority of patients with indication for tonsil surgery presented with 3–5 tonsillitis episodes in the last 12 months. The proportion of patients with < 3 episodes decreased, whereas the proportion of patients with ≥ 6 episodes increased (p < 0.001). The surgical rate per 100,000 population in regard of the number of tonsillitis episodes are summarized in Table 2. The highest surgical rates were seen in all three years for patients with 3–5 episodes. The surgical rates for patients with < 3 episodes were higher than for patients with ≥ 6 episodes in 2011 and 2015. This changed in 2019. Here, the surgical rates for patients with ≥ 6 episodes were for the first time higher than for patients with < 3 episodes (Fig. 3).

**Table 3:** Tonsillitis episodes treated with antibiotics in patients who underwent tonsil surgery (n = 1382).

Parameter	All		2011		2015		2019		p
	n	%	n	%	n	%	n	%	
All	1383	100	625	100	510	100	248	100	** < 0.001**
< 3 episodes last 12 months	276	20	133	21.3	105	20.6	38	15.3	
3–5 episodes last 12 months	862	62.3	404	64.6	337	66.1	121	48.8	
≥ 6 episodes last 12 months	245	17.7	88	14.1	68	13.3	89	35.9	

	Mean ± SD	Mean ± SD	Mean ± SD	Mean ± SD					
Tonsillitis episodes last 12 months, n	3.99** ± **2.01	3.82** ± **1.89	3.79** ± **1.89	4.79** ± **2.32	** < 0.001**				

Significant p-values (p < 0.05) in bold.

*SD* standard deviation.

### Comparison of patients who have undergone surgery with < 3 tonsillitis episodes versus 3-5 tonsillitis episodes versus ≥ 6 tonsillitis episodes in last 12 months

Table 4 gives an overview about the results of the comparison. Patients with 3–5 or ≥ 6 tonsillitis episodes were older compared to the patients with < 3 episodes; p < 0.001). The proportion of patients with < 3 or 3–5 episodes decreased over the years, whereas the proportion of patients with ≥ 6 episodes increased (p < 0.001). The number of patients undergoing tonsillotomy instead of tonsillectomy as well as with tonsil surgery only one side were very low. Nevertheless, patients with < 3 episodes received more frequently a tonsillotomy or single sided surgery than patients with 3–5 or ≥ 6 tonsillitis episodes (p < 0.001 and p = 0.001, respectively). Other differences were not seen.

**Table 4:** Comparison of operated RAT patients with 1–5 tonsillitis episodes versus ≥ 6 tonsillitis episodes in last 12 months (N = 1383).

Parameter	RAT with < 3 tonsillitis episodes in last 12 months		RAT with 3–5 tonsillitis episodes in last 12 months		RAT with ≥ 6 tonsillitis episodes in last 12 months		p
	n	%	n	%	n	%	
All	276	100	862	100	245	100	
Gender							0.231
Male	120	43.5	326	37.8	99	40.4	
Female	156	56.5	536	62.2	146	59.6	
Year							** < 0.001**
2011	133	48.2	404	46.9	88	35.9	
2015	105	38.0	337	39.1	68	27.8	
2019	38	13.8	121	14.0	89	36.3	
Prior tonsil surgery							0.208
Yes	12	4.3	20	2.3	7	2.9	
No	264	95.7	842	97.7	238	97.1	
Prior adenoidectomy							0.091
Yes	26	9.4	120	13.9	26	10.6	
No	250	90.6	742	86.1	219	89.4	
Tonsillectomy/tonsillotomy							** < 0.001**
Tonsillectomy	263	95.3	853	99.0	242	98.8	
Tonsillotomy	13	4.7	9	1.0	3	1.2	
Additional adenoidectomy							0.546
Yes	0	0	2	22.2	0	0.0	
No	276	100	860	99.8	245	100.0	
Side of surgery							**0.001**
One side	9	3.3	5	0.6	2	0.8	
Both sides	267	96.7	857	99.4	243	99.2	

	Mean ± SD	Mean ± SD	Mean ± SD				
Age, years*	22.0 ± 12.8	25.3 ± 12.3	24.6 ± 11.6	** < 0.001**			

Significant p-values (p < 0.05) in bold.

*NA* not applicable, *SD* standard deviation.

*ANOVA with post-hoc Bonferroni correction: no difference between 2015 and 2019.

### Multivariate analysis of changes between 2011, 2015 and 2019

A multinomial logistic regression analysis of patients’ characteristics changing from the patients’ cohort in year 2011 as reference to the cohorts in 2015 and 2019 (Table 5). Compared to the year 2011, the age of the patients undergoing surgery was higher in 2015 (OR 1.024; CI 1.014–1.034; p < 0.001) and higher in 2019 (OR 1.030: CI 1.017–1.043; p < 0.001). Compared to 2011, the number of tonsillitis episodes was not higher in 2015, but in 2019 (OR 1.273; CI 1.185–1.367; p < 0.001). In addition, the probability to receive a tonsillotomy instead of a tonsillectomy was not higher in 2015 compared to 2011, but for 2019 compared to 2011 (OR 6.173; CI 2.222–17.241; p < 0.001).

**Table 5:** Multinomial logistic regression analysis of patients’ characteristics changing from the patients’ cohort in 2011 (reference) to the cohorts in 2015 and 2019.

Patients’ characteristics	2011	2015		2019	
	Reference	OR (95% CI)	p	OR (95% CI)	p
Age	1	1.024 (1.014–1.034)	** < 0.001**	1.030 (1.017–1.043)	** < 0.001**
Number of tonsillitis episodes last 12 months	1	1.014 (0.950–2.082)	0.681	1.273 (1.185–1.367)	** < 0.001**
Tonsillotomy instead of tonsillectomy	1	1.851 (0.675–5.076)	0.232	6.173 (2.222–17.241)	** < 0.001**

Significant p-values (p < 0.05) in bold.

*OR* odds ratio, *CI* confidence interval.

## Discussion

There was a very strict paradigm shift in Germany in 2015 for indication for tonsil surgery in patients with RAT from no defined criteria before to very hard nevertheless somehow arbitrary criteria in the very first German tonsillitis guideline. This might on the one hand explain why in the present study cohort even 2019 still only 36% of the RAT patients had ≥ 6 tonsillitis episodes prior to tonsil surgery. On the other hand, the number of surgeries for RAT decreased by 59% from 2011 to 2019 in Thuringia. This shows that significantly fewer patients were presented to the clinics for surgery. It is likely that referring physicians presented fewer patients with a low number of tonsillitis episodes. This speaks for an increasing guideline adherence over the years. For the first time, we could also present surgical rates in relation to the number of tonsillitis episodes. Most patients had in all three analyzed years 3–5 tonsillitis episodes, but the groups of patients with ≥ 6 episodes nearly caught up the group with 3–5 tonsillitis episodes in 2019. An older study from the United Kingdom for the years 2005 to 2016 revealed that 0.4%, 1.0%, 3.2%, and 8.9% of the patients consulted in general practice with 1, 2, 3–4, and 5–6 sore throat episodes, respectively, underwent tonsillectomy^9^. Such a linkage to data from general practitioners was not possible in the present study. Nevertheless, it can be concluded that (1) the number of tonsil surgeries in general have decreased, (2) the proportion of patients with ≥ 6 episodes has increased, whereas the numbers for patients with < 6 episodes have decreased after introduction of the German tonsillitis guideline, and (3) this shift went along with an increase of the age of patient when they underwent surgery. This age shift has been seen also in the United Kingdom after implementation of the SIGN criteria^10^. At least the dramatic reduction of tonsillectomies after implementation of the SIGN guidelines was also seen in the United Kingdom^11,12^. In contrast, countries with lower cut-off for indication of tonsil surgery like in Scandinavia with ≥ 3 episodes report constant higher number of surgery^13,14^.

A further increase of guideline adherence in daily routine can be expected with continuous medical education concerning the guideline^15^, and mainly by increasing evidence that RAT patients in any age group with ≥ 6 episodes really profit more from tonsil surgery than patients with less severe symptoms^16–18^. In this regard, the recently published results of the Tonsillectomy IN Adults (NATTINA) are very important^19^. NATTINA was a multicenter, randomized, controlled trial for adults with recurrent tonsillitis to compare the clinical and cost-effectiveness of tonsillectomy versus conservative management in the United Kingdom. The study showed that immediate tonsillectomy was clinically effective and cost-effective in adults with RAT defined by ≥ 7 clinically significant sore throat episodes in the preceding year (UK guideline for tonsillectomy). This should help to set the doctors on a clear cut-off for the tonsil surgery indication. Therefore, it is also to be expected for Germany that the group fulfilling > 6 tonsillitis episodes before undergoing tonsil surgery will rise.

Although evidence was low, the German tonsillitis guideline of 2015 already recommended tonsillotomy as an adequate tonsil surgery technique for RAT based on the same episode criteria as for tonsillectomy. The present study has shown that the proportion of tonsillotomy compared to tonsillectomy still was—although increasing—very low in 2019 in Thuringia with 3.6% of all tonsil surgeries. Probably, more hard data are needed that tonsillotomy is also adequate to treat RAT. In this regard, several ongoing trials are of interest. The TOTO study is a German multicenter randomized non-inferiority trial comparing tonsillotomy versus tonsillectomy in patients with ≥ 6 episodes of tonsillitis^20^. A Finish multicenter, randomized, parallel-group clinical study compares tonsillectomy and tonsillotomy (TT), to watchful waiting^21^. Finally, the FINITE study is a prospective, randomized three-arm comparing three different surgical methods being extracapsular monopolar tonsillectomy versus intracapsular microdebrider tonsillectomy versus intracapsular coblation tonsillectomy in the treatment of adult patients in Finland^22^.

The present study has some limitations. The amount of patients without documented number of tonsillitis episodes was high. A selection bias cannot be excluded. The real shift from tonsillectomy to tonsillotomy in 2019 might be higher, as especially for tonsillotomy patients the number of episodes often was not adequately documented. This documentation problem is well known^11^. Some tonsillotomies might been performed as outpatient procedures, but this rate is low in Thuringia (< 2%) since many years. Regularly repeated education, quick references guides, and check lists might help in clinical routine to improve the documentation^12^. Furthermore, it can be that some of the excluded patients had an indication for tonsil surgery based on counting episodes without requiring antibiotics. Due to the exclusion of a relevant amount of patients, the true surgical rates for RAT might be underestimated. In prior study, we calculated for 2012 an incidence of tonsil surgery for RAT with 36.9/100,000 population, i.e., higher than in the present study^23^. Even an optimal documentation of the episodes would not completely solve the problems with inaccuracies. Just as problematic is the accuracy of proof of the antibiotic treatments. At the moment, this is only based on patient information. The verification that the information is true appears not to play a major role when tonsillectomy is indicated^24^. In the current process of the revision of the tonsillitis guideline, one must ask if the passage in the recommendations regarding the tonsillitis episodes “treated with antibiotics” cannot be deleted.

In conclusion, this population-based analysis demonstrated that the introduction of the German tonsillitis guideline was followed by a behavioral change in the indication of tonsil surgery for RAT patients. Nevertheless, a relevant amount of patients with moderate tonsillitis severity still receives tonsil surgery. More data are needed whether tonsillotomy is equivalent to tonsillectomy when surgery is indicated.

### Supplementary Information

Supplementary Table S1.

## Data Availability

The data presented in this study are available on request from the corresponding author.
