# Supplementary material for: Effect of the German tonsillitis guideline on indication for tonsil surgery in patients with recurrent acute tonsillitis: a population-based study
Source: Sci Rep. 2023 Oct 17;13:17612. doi: 10.1038/s41598-023-44661-y (PMC10582004; doi:10.1038/s41598-023-44661-y)
Supplement: Supplementary file 1 — Supplementary Table S1. [file 41598_2023_44661_MOESM1_ESM.docx]

**Effect of the German tonsillitis guideline on indication for tonsil surgery in patients with recurrent acute tonsillitis: a population-based study**

Valerie Betz, Daniel Boeger, Jens Buentzel, Kerstin Hoffmann, Peter Jecker, Holger Kaftan, Andreas Mueller, Gerald Radtke, Katharina Geißler, Orlando Guntinas-Lichius

**Supplement Table 1**

| **Supplement Table 1.** Comparison of included and excluded patients with diagnosis of recurrent acute tonsillitis (RAT). | | | |
| --- | --- | --- | --- |
| **Parameter** | **RAT with documented number of tonsillitis episodes** | **RAT without documented number of tonsillitis episodes** | **p** |
|  | **n = 1398** | **n = 611** |  |
| Gender |  |  | 0.726 |
| Male | 547 | 234 |  |
| Female | 851 | 377 |  |
| Year |  |  | **0.007** |
| 2011 | 626 | 279 |  |
| 2015 | 518 | 254 |  |
| 2019 | 254 | 78 |  |
| Reason against surgery |  |  | **<0.001** |
| Patient had surgery | 1383 | 544 |  |
| Contraindication | 1 | 2 |  |
| Patient declined surgery | 1 | 0 |  |
| No indication | 13 | 60 |  |
| Prior tonsil surgery, no indication for revision surgery | 0 | 5 |  |
| Setting |  |  | **<0.001** |
| Inpatient | 1389 | 576 |  |
| Outpatient | 9 | 23 |  |
| Surgery |  |  | **<0.001** |
| Yes | 1383 | 544 |  |
| No | 15 | 67 |  |
| Tonsillectomy/Tonsillotomy* |  |  | **<0.001** |
| Tonsillectomy | 1356 | 485 |  |
| Tonsillotomy | 25 | 41 |  |
| Additional adenoidectomy |  |  | **<0.001** |
| No | 1381 | 525 |  |
| Yes | 2 | 17 |  |
| Side of surgery |  |  |  |
| One side | 16 | 31 |  |
| Both sides | 1367 | 511 |  |
|  | **Mean±SD** | **Mean±SD** |  |
| Age, years | 23.8±12.4 | 28.6±17.6 | **<0.001** |
|  |  |  |  |

*±adenoidectomy; SD = standard deviation; SD = standard deviation; significant p-values (p<0.05) in bold.
